# Characterization of New Polyphenolic Glycosidic Constituents and Evaluation of Cytotoxicity on a Macrophage Cell Line and Allelopathic Activities of *Oryza sativa*

**DOI:** 10.3390/molecules23081933

**Published:** 2018-08-02

**Authors:** Ill-Min Chung, Chang Kwon, Yeonju An, Mohd Ali, Hannah Lee, Jung-Dae Lim, Soyeon Kim, Yujin Yang, Seung-Hyun Kim, Ateeque Ahmad

**Affiliations:** 1 Department of Applied Bioscience, College of Life and Environmental Science, Konkuk University, Seoul 05029, Korea; imcim@konkuk.ac.kr (I.-M.C.); chang794@konkuk.ac.kr (C.K.); ayj3043@konkuk.ac.kr (Y.A.); hellosy1@konkuk.ac.kr (S.K.); jin9031@konkuk.ac.kr (Y.Y.); kshkim@konkuk.ac.kr (S.-H.K.); 2 Department of Pharmacognosy and Phytochemistry, Hamdard University, New Delhi 110062, India; maliphyto@gmail.com; 3 Department of Herbal Medicine Resource, Kangwon National University, Samcheok 25949, Korea; dlgkssk1009@naver.com (H.L.); ijdae@kangwon.ac.kr (J.-D.L.); 4 Process Chemistry and Technology Department, CSIR-Central Institute of Medicinal and Aromatic Plants, Lucknow 226015, India

**Keywords:** *Oryza sativa* L., *Gramineae*, rice leaves and straw, new chemical constituents, cytotoxicity test, allelopathic activities, *E. oryzicola*

## Abstract

Four new constituents, as 5, 7-dihydroxy-4′-methoxyflavonol-3-*O*-*β*-d-arabinopyranosyl-(2′′→1′′′)-*O*-*β*-d-arabinopyrnosyl-2′′′-*O*-3′′′′, 7′′′′-dimethylnonan-1′′′′-oate (**1**), 5-hydroxy-7, 4′-dimethoxyflavone-5-*O*-*α*-d-arabinopyranosyl-(2"→1′′′)-*O*-*α*-d-arabinopyranosyl-2′′′-*O*-3′′′′, 7′′′′-dimethylnonan-1′′′′-oate (**2**), 5-hydroxy-7, 4′-dimethoxyflavone-5-*O*-*β*-d-arabinofuranosyl-(2"→1′′′)-*O*-*β*-d-arabinopyranosyl-2′′′-*O*-lanost-5-ene (**3**) and 4′,4′′-diferuloxy feruloyl-*O*-*α*-d-arabinopyranosyl-(2a→1b)-*O*-*α*-d-arabinopyranosyl-(2b→1c)-*O*-*α*-d-arabinopyranosyl-(2c→1d)-*O*-*α*-d-arabinopyranosyl-(2d→1e)-*O*-*α*-d-arabinopyranosyl-2e-3′′′, 7′′′-dimethylnonan-1′′′-oate (**4**), along with three known compounds (**5**–**7**) were isolated from *Oryza sativa* leaves and straw. The structures of new and known compounds were elucidated by 1D (^1^H and ^13^C NMR) and 2D NMR spectral methods, *viz*: COSY, HMBC, and HSQC aided by mass techniques and IR spectroscopy. The cytotoxicity of these constituents was assessed by using (RAW 264.7) mouse macrophage cell line, and allelopathic effects of compounds (**1**–**7**) on the germination and seedling growth characteristics such as seedling length and root length of barnyardgrass (*Echinochloa oryzicola*) were evaluated. Significant inhibitory activity was exhibited by compounds comprising flavone derivatives such as (**1**–**3**) on all of seed germination characteristics. The allelopathic effect of flavone derivatives were more pronounced on seedling length and root length than the germination characteristics. The higher concentration of flavone derivatives showed stronger inhibitory effects, whereas the lower concentrations showed stimulatory effects in some cases.

## 1. Introduction

Rice (*Oryza sativa* L.) is the major staple food in Asia and generally exists as two types, white hulled and colored hulled. The most common type (85%) is white-hulled rice. The germination of rice is of great agricultural importance and has long been known to be influenced by compounds present in the seed coat (hull) [1,2]. The compounds momilactones A and B from rice hulls cause germination and growth inhibition in the rice roots [3,4,5]. They are also found in rice leaves and rice straw as phytoalexins [6,7,8]. Identification of allelopathic compounds, including momilactones A and B, from rice straw and husks and their biological activities have been reported [9,10,11,12,13,14]. Flavonoid glycosides from leaves and straw of *O*. *sativa* and their effects of cytotoxicity on a macrophage cell line and allelopathic on weed germination have been reported [15].

**Figure 1:**
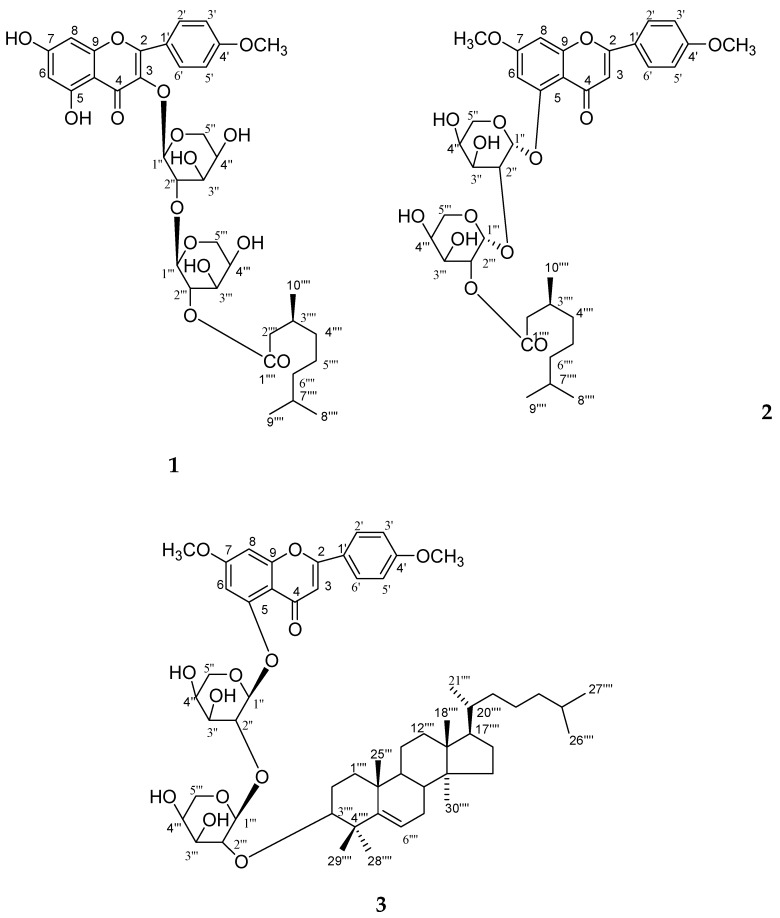
Chemical structure of compounds **1**–**3**.

**Figure 2:**
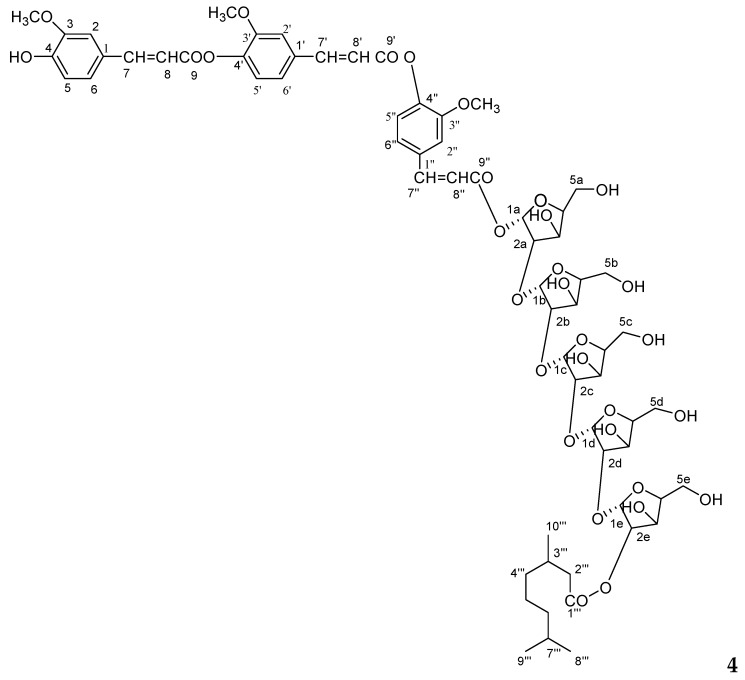
Chemical structure of compound **4**.

**Figure 3:**
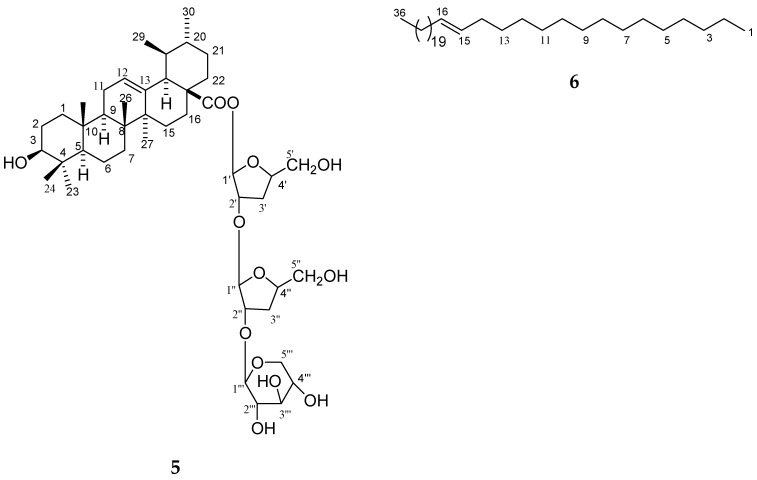

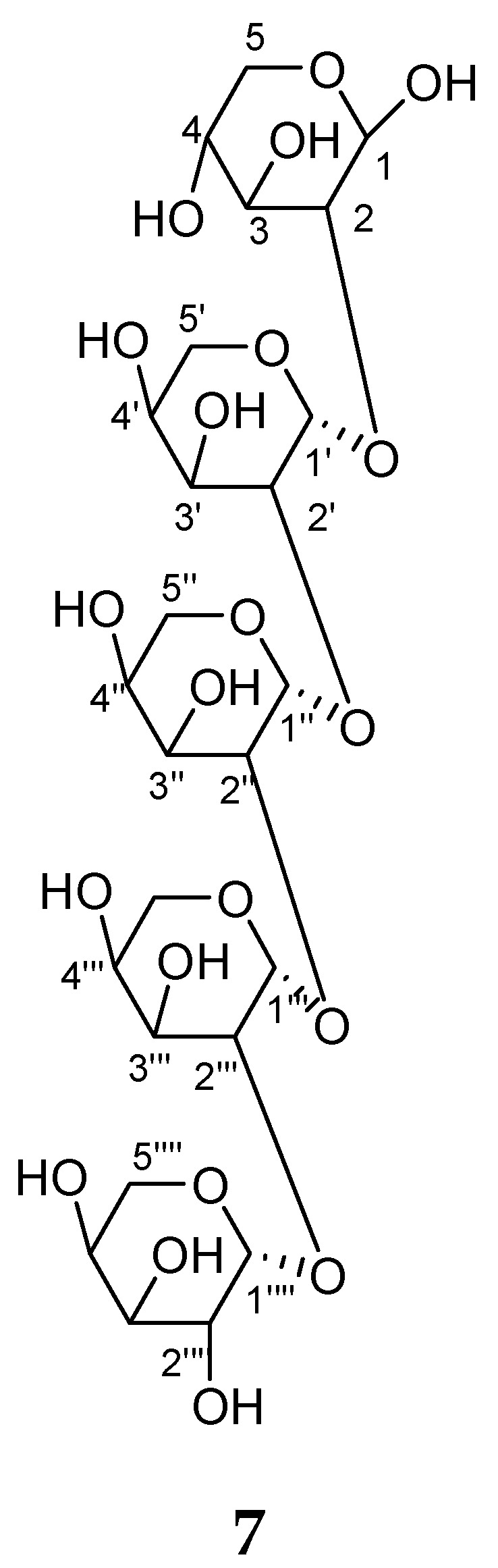
Chemical structure of compounds **5**–**7**.

Plant–plant interactions can be negative and may depend on concentrations of flavonoids [16,17,18]. Several studies have shown that a few flavones and other types of compounds are also potent allelochemicals from rice. Allelopathic activity is mainly based on inhibiting germination and growth of other plants seedlings and it can be successfully exploited for weed management. Although many studies have attempted to evaluate the allelopathic activity of various rice parts and to analyze allelochemicals in rice, little information is available on the correlation between bioassays such as cytotoxicity and anti-oxidative activity and one of the potential allelochemicals from straw and leaves of *O*. *sativa*. The objective of this study is the identification of new and known constituents along with biological activities of compounds (**1**–**7**) from *O*. *sativa* of straw and leaves. The identified four new compounds as 5, 7-dihydroxy-4′-methoxyflavonol-3-*O*-*β*-d-arabinopyranosyl-(2′′→1′′′)-*O*-*β*-d-arabinopyrnosyl-2′′′-*O*-3′′′′, 7′′′′-dimethylnonan-1′′′′-oate (**1**), 5-hydroxy-7, 4′-dimethoxyflavone-5-*O*-*α*-d-arabinopyranosyl-(2′′→1′′′)-*O*-*α*-d-arabinopyranosyl-2′′′-3′′′′, 7′′′′-dimethylnonan-1′′′′-oate (**2**), 5-hydroxy-7, 4′-dimethoxyflavone-5-*O*-*β*-d-arabinofuranosyl-(2"→1′′′)-*O*-*β*-d-arabinopyranosyl-2′′′-*O*-lanost-5-ene (**3**) and 4′,4′′-diferuloxy feruloyl-*O*-*α*-d-arabinopyranosyl-(2a→1b)-*O*-*α*-d-arabinopyranosyl-(2b→1c)-*O*-*α*-d-arabinopyranosyl-(2c→1d)-*O*-*α*-d-arabinopyranosyl-(2d→1e)-*O*-*α*-d-arabinopyranosyl-2e-3′′′, 7′′′-dimethylnonan -1′′′-oate (**4**) and three known compounds, ursolic acid 28-*O*-*β*-d-xylofuranosyl-(2′→1′′)-*O*-*β*-d-xylofuranosyl-(2′′→1′′′)-*O*-*β*-d-xylopyranoside (**5**), *n*-hexatriacont-15-ene (**6**) and as *β*-d-xylopyranosyl-(2→1′)-*O*-*α*-d-arabinopyranosyl-(2′→1′′)-*O*-*α*-d-arabinopyranosyl-(2′′→1′′′)-*O*-*α*-d-arabinopyranosyl-(2′′′→1′′′)-*O*-*α*-d-arabinopyranosyide (**7**). The cytotoxicity of the new and known compounds (**1**–**7**, Figure 1, Figure 2 and Figure 3) was evaluated in a macrophage cell line RAW 264.7 by using an MTT assay and evaluated for their allelopathic effect on barnyardgrass (*E. oryzicola*), and characterization of weed seed germination and morphology was accomplished by treatment with different concentrations of the purified natural products. The objective of the present investigation was to report natural products and biological activities of compounds (**1**–**7**) from leaves and straw of *O*. *sativa*.

## 2. Results and Discussions

**Figure 4:**
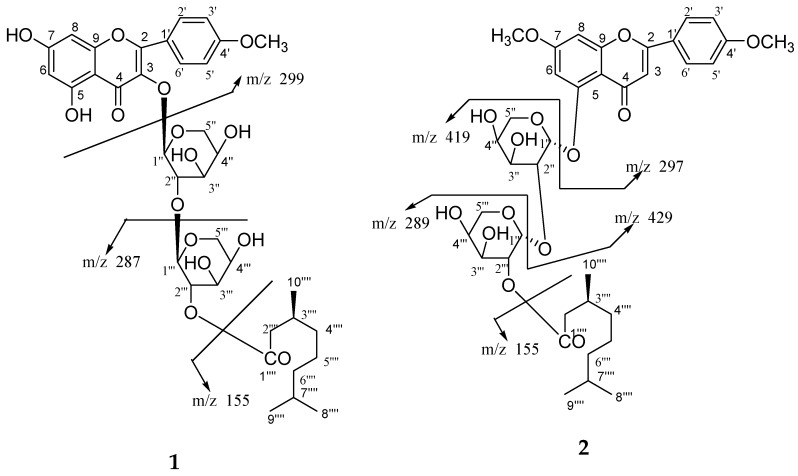

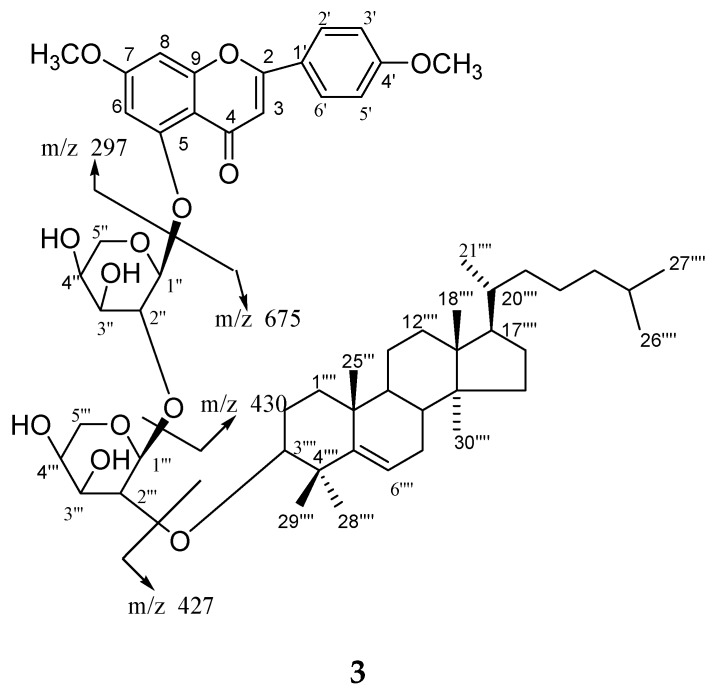
Fragmentation patterns of compounds **1**–**3**.

**Figure 5:**
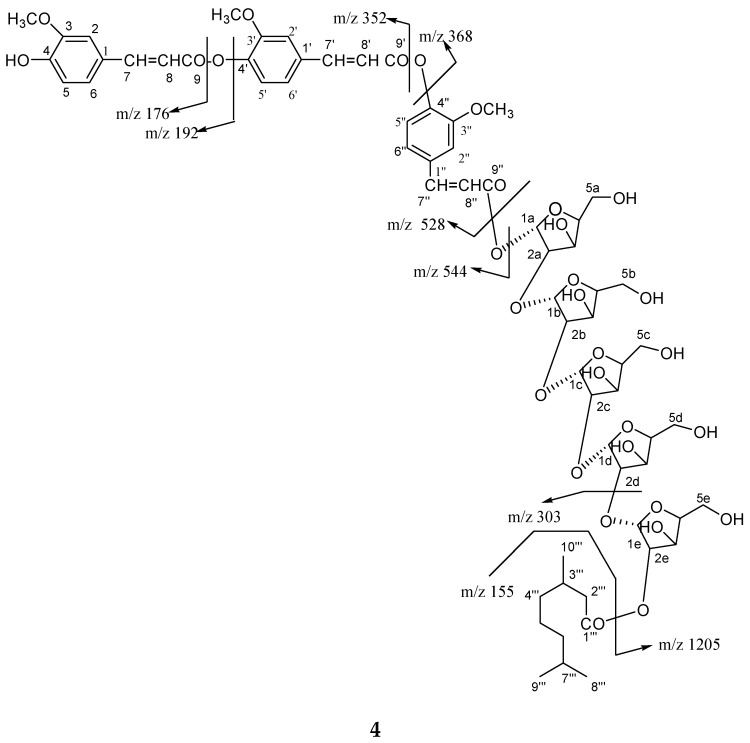
Fragmentation pattern of compound **4**.

The methanol extract of leaves and straw of *Oryza sativa* was column chromatographed and seven compounds (**1**–**7**) were obtained. Their structures were elucidated on the basis of spectroscopic data (as shown in Figure 1, Figure 2, Figure 3, Figure 4 and Figure 5 and Table 1, Table 2, Table 3, Table 4 and Table 5). The details of structure elucidation are described in the results and discussion.

Compound **1** was obtained as a yellow powder from hexane/EtOAc (6:4, *v*/*v*) eluents (Figure 1). It tested positive with the ferric chloride test for a phenolic moiety. The UV absorption maxima, 270, 309, and 336 nm, were typical for a flavonoid [19,20,21,22,23]. Upon addition of sodium acetate, there was no UV absorption band shift, suggesting a bound C-7 hydroxyl group. The characteristic UV absorption maxima were in agreement with those in the literature [19]. A bathochromic shift of 40 nm upon addition of AlCl_3_ and AlCl_3_/HCl indicated the presence of a chelated hydroxyl group at C-5 [19]. The IR spectrum displayed absorption bands for hydroxyl groups (3450, 3358, 3271 cm^−1^), an ester function (1721 cm^−1^), carbonyl groups (1685 cm^−1^), and aromatic rings (1602, 1514 cm^−1^). On the basis of the ^13^C NMR and mass spectra, the molecular ion peak of a **1** was assigned as *m*/*z* 719 and was consistent with the molecular formula of a flavonol triglycosidic ester C_36_H_47_O_15_. High resolution ESI/FTMS provided the exact mass of the protonated molecular ion, which was consistent with this molecular formula. The fragmentation pattern of compound **1** is shown in Figure 4.

The ^1^H NMR spectrum of **1** indicated a flavonoid moiety, as it displayed two one-proton meta coupled singlets at *δ* 6.25 and 6.31 which were ascribed to H-6 and H-8 protons in a 5,7-oxygenated ring, and four ortho-coupled double doublets and doublets at *δ* 7.65 (*J* = 7.5, 1.5 Hz), 6.81 (*J* = 7.5, 2.0 Hz), 6.78 (*J* = 8.5, 2.0 Hz) and 7.43 (*J* = 8.5 Hz) ascribed to H-2′, H-6′, H-5′, and H-6′ suggesting a 4′-oxygenated substitution pattern in ring B; a meta-coupled AX system corresponding to the H-6 and H-8 protons in ring A and ortho-coupled protons characteristic of an AA′XX′ spin system of a para-substituted phenyl ring were found for ring B. 

The sugar units in **1** were identified as *β*-d-arabinopyranose by analysis of the coupling constant of anomeric signals of sugar protons as one-proton doublets at *δ* 5.43 (*J* = 7.1 Hz, H-1′′) and 4.96 (*J* = 7.2 Hz, H-1′′′), respectively. The remaining sugar protons appeared as multiplets at *δ* 4.96−3.29 for H-2′′, H-3′′, H-4′′ and H-2′′′, H-3′′′, H-4′′′. The methylene protons in sugars appeared as double doublets *δ* 3.30, 3.29 (*J* = 3.0, 3.0, H_2_-5′′a, H_2_-5′′b) and doublet 3.59 (*J* = 11.5 Hz, H_2_-5′′′). Three proton doublets at *δ* 0.96 (*J* = 7.0 Hz), 1.01 (*J* = 8.0 Hz), and 1.28 (*J* = 6.5 Hz) were assigned to methyl protons in ester function moiety for C-8′′′′, C-9′′′′ and C-10′′′′ protons. A double doublet at *δ* 2.03 (*J* = 8.5 Hz), 2.07 (*J* = 9.0 Hz) and three multiplets at *δ* 1.32, 1.18, 1.13 were ascribed to methylene H_2_-2′′′′, H_2_-4′′′′, H_2_-5′′′′ and H_2_-6′′′′ and methine protons at *δ* 1.89 and 1.58 for H-3′′′′ and H-7′′′′ in ester moiety protons. A broad three-proton signal at *δ* 3.36 was due to the methoxy protons.

The ^13^C NMR spectrum of **1** showed signals for a C-4 flavone carbonyl carbon at *δ* 175.32; other flavone carbons resonated between *δ* 161.01 and 94.35, anomeric carbons at *δ* 104.89 (C-1′′) and 93.96 (C-1′′′), ester carbon at *δ* 171.24 (C-1′′′′), and ester methyl carbons at *δ* 20.60 (C-8′′′′), 20.83 (C-9′′′′), and 28.75 (C-10′′′′). The ^1^H and ^13^C NMR spectroscopic data of the flavonol unit were compared with reported spectroscopic values for these compounds [19,20,21,22,23]. The appearance of the C-3 signal at *δ* 131.27 in the ^13^C NMR spectrum supported the attachment of the sugar moiety at this carbon [19,20,21,22,23]. The presence of the sugar ^1^H NMR signals of H-1′′, H-2′′, H-3′′, and H-4′′ between *δ* 5.43 and 3.83, and the ^13^C NMR signals at *δ* 104.89 (C-1′′), 89.12 (C-2′′), 77.88 (C-3′′), and 73.81 (C-4′′) in the deshielded region suggested (2→1) linkages of the sugar units. Furthermore, the ^1^H and ^13^C NMR spectroscopic data (Table 1) exhibited the presence of *β*-d-arabinopyranose by analysis of the coupling constants (*J*_1′′ 2′′ and 1′′′ 2 ′′′_ = 7.1, 7.2) of the anomeric signals of sugar protons.

**Figure 6:**
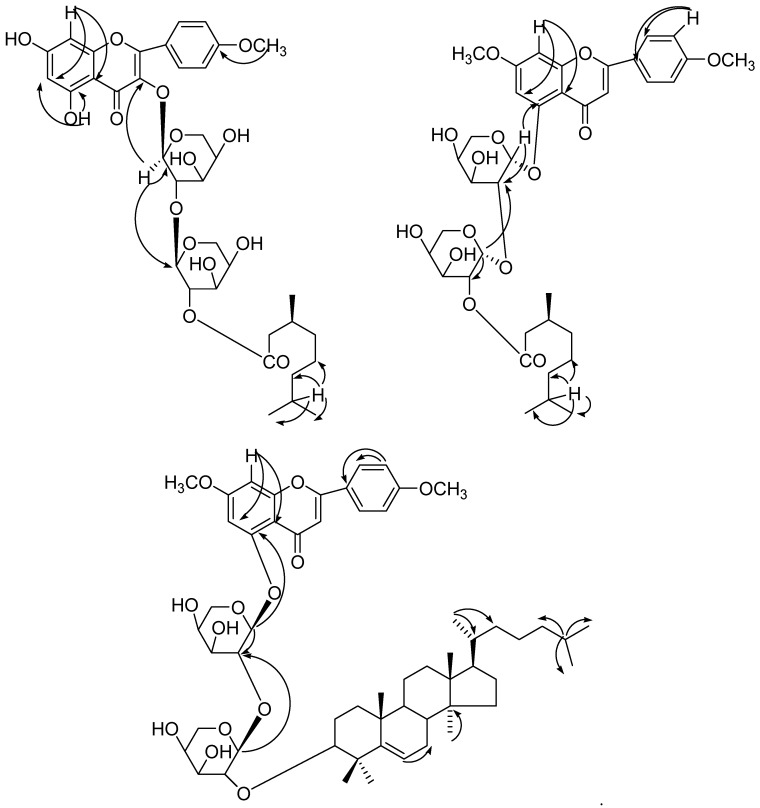
HMBC correlation of compounds **1**–**3**.

The ^1^H-^1^H COSY spectrum of **1** showed correlations of H-6 with H-8; H-2′ with H-3′ and H-6′; H-5′ with H-6′; H-2′′ with H-1′′, H-3′′ and H-1′′′; H-2′′′ with H-1′′′; and H_3_-9′′′′ with H-7′′′′ and H-8′′′′. The appearance of the C-3 signal at *δ*131.27 in the ^13^C NMR spectrum supported the attachment of the sugar moiety at this carbon [24,25]. The HMBC spectrum of **1** exhibited interaction of H-1′′ with C-3 and C-1′′′; H-1′′′ with C-1′′, confirming (2→1) sugar units and attachment of the innermost sugar at C-3. H-8 interacted with C-6 and C-10 and H-5 correlated with C-6 also; H-7 correlated with C-8′′′′, C-9′′′′ and C-6′′′′ (Figure 6). The HSQC correlations were used to assign all protons and carbon atoms with the corresponding units and established possible links with other parts of the molecule; some important correlation in the HSQC spectrum are H-1′′ with C-1′′; H-1′′′ with C-1′′′. On the basis of the analysis of the spectroscopic data and the chemical reactions, the structure of **1** was established as 5, 7-dihydroxy-4′-methoxyflavonol-3-*O*-*β*-d-arabinopyranosyl-(2′′→1′′′)-*O*-*β*-d-arabinopyrnosyl-2′′′-*O*-3′′′′, 7′′′′-dimethylnonan-1′′′′-oate, which is a new compound.

Compound **2** was obtained as a light yellow powder from hexane/EtOAc (6:4, *v*/*v*) eluants. The UV absorption maxima at 265, 302, and 331 were typical for flavonoids [19,20,21,22,23]. The characteristic UV absorption maxima were in agreement with those in the literature [19]. The IR spectrum displayed absorption bands for hydroxyl groups (3451, 3369, 3283 cm^−1^), ester function (1720 cm^−1^), carbonyl groups (1607, 1635 cm^−1^), aromatic ring (1513 cm^−1^), and aliphatic chain (836 cm^−1^). On the basis of the ^13^C NMR and mass spectra, the molecular ion peak of **2** was consistent with the molecular formula of a flavonol diglycosidic ester *m*/*z* 717, C_37_H_49_O_14_. High resolution ESI/FTMS provided the exact mass of the protonated molecular ion peak with this molecular formula. The fragmentation pattern of compound **2** is shown in Figure 4.

The ^1^H NMR spectrum of **2** indicated a flavone moiety as it displayed two one-proton meta-coupled singlets at *δ* 6.43, 6.64 and four ortho-coupled doublets at *δ* 7.15 (*J* = 8.5 Hz), 6.81 (*J* = 8.5 Hz), 6.77 (*J* = 10.5 Hz) and 7.13 (*J* = 10.5 Hz), which were assigned to H-6, H-8, H-2′, H-3′, H-5′ and H-6′ protons suggesting a 4′-oxygenated substitution pattern in ring B; and suggesting an AA′ BB′ spin system. The sugar units in **2** were identified as *α*-d-arabinopyranoside by the analysis of the coupling constant of the anomeric signals of sugar protons at *δ* 5.01 (*J* = 6.5 Hz), 4.93 (*J* = 5.0 Hz). The presence of the sugar ^1^H NMR signals of H-2′′ at *δ*3.88 and the ^13^C NMR signal 78.51 in the deshielded region suggested (2→1) linkage of the sugar units. Three doublets at *δ* 0.98 (*J* = 6.6 Hz), 0.93 (*J* = 6.4 Hz) and 1.16 (*J* = 6.5 Hz) were ascribed for three methyl protons for Me-8′′′′, Me-9′′′′ and Me-10′′′′. Two broad singlets three protons each at *δ* 3.29, 3.34 were assigned for C-7 and C-4′ methoxy protons.

The ^13^C NMR spectrum of **2** showed signals for a C-4 flavone carbonyl carbon at *δ* 183.94; other flavone carbons resonated between *δ* 164.75 and 96.32, anomeric carbons at *δ* 105.22 (C-1′′) and 96.32 (C-1′′′), ester carbon at *δ* 171.26 (C-1′′′′), and ester methyl carbons at *δ* 26.45 (C-8′′′′), 26.07 (C-9′′′) and 30.14 (C-10′′′′). The ^1^H and ^13^C NMR spectroscopic data of the flavonol unit were compared with reported spectroscopic values for these compounds [19,20,21,22,23]. The presence of the sugar ^1^H NMR signals of H-1′′, H-2′′, H-3′′ and H-4′′ between *δ* 5.43 and 3.83, and the ^13^C NMR signals at *δ* 104.89 (C-1′′), 89.12 (C-2′′), 77.88 (C-3′′) and 73.81 (C-4′′) in the deshielded region suggested (2→1) linkages of the sugar units. Furthermore, the ^1^H and ^13^C NMR spectroscopic data Table 2 exhibited the presence of *α*-d-arabinopyranose by analysis of the coupling constants (*J*_1′′ 2′′ and 1′′′ 2 ′′′_= 6.5, 5.0) of the anomeric signals of sugar protons [22,23,24,25,26].

The ^1^H-^1^H COSY spectrum of **2** showed correlations of H-6 with H-8; H-2′ with H-3′ and H-6′; H-5′ with H-6′; H-2′′ with H-1′′, H-3′′ and H-1′′′; H-2′′′ with H-1′′′; and H-7′′′ with H-6′′′′, H-7′′′′, H-8′′′′ and. H-9′′′′. The HMBC spectrum of **2** exhibited interaction of H-1′′ with C-5 and C-2′′; H-1′′′ with C-2′′ and C-′′′3 confirming (2→1) sugar units and attachment of the innermost sugar at C-5; and H-7 correlated with C-8′′′′, C-9′′′′ and C-6′′′′ and H-8 interacted with C-6 and C-10 also (Figure 6). The arabinopyranosyl residue was located at the C-5 position of the flavones skeleton according to long-range HMBC correlation between C-5 at *δ* 158.85 and the anomeric H-1′′ at *δ* 5.01 was compared with literature data [26]. The HSQC correlations were used to assign all protons and carbon atoms with the corresponding units and established possible links with other parts of the molecule; some important correlation in the HSQC spectrum are H-1′′ with C-1′′; H-1′′′ with C-1′′′. On the basis of the analysis of the spectroscopic data and the chemical reactions, the structure of **2** was established as 5-hydroxy-7, 4′-dimethoxyflavone-5-*O*-*α*-d-arabinopyranosyl-(2′′→1′′′)-*O*-*α*-d-arabinopyranosyl-2′′′, 3′′′′, 7′′′′-dimethylnonan-1′′′′-oate, which is a new compound.

Compound **3** was obtained a light yellow viscous mass from hexane/EtOAc (2:8, *v*/*v*) eluants. The IR spectrum displayed absorption bands for hydroxyl groups (3450, 3355, 3228 cm^−1^), carbonyl groups (1690, 1635 cm^−1^), aromatic ring (1525 cm^−1^) and aliphatic chain (835 cm^−1^). On the basis of the ^13^C NMR and mass spectra, the molecular ion peak of **3** was consistent with the molecular formula of a flavonol monoglycosidic ester *m*/*z* 973, C_57_H_81_O_13_. High resolution ESI/FTMS provided the exact mass of the protonated molecular ion peak with this molecular formula. The fragmentation pattern of compound **3** is shown in Figure 4.

The ^1^H NMR spectrum of **3** indicated a flavone moiety as it displayed two one-proton meta-coupled doublets at *δ* 6.20 (*J* = 2.0 Hz), 6.31 (*J* = 2.0 Hz), and four ortho-coupled double doublets at *δ* 7.41 (*J* = 9.0, 3.5 Hz) and 6.75 (*J* = 2.0, 9.0 Hz), 6.72 (*J* = 2.0, 8.5 Hz), and 7.05 (*J* = 3.0, 8.5 Hz), were assigned to H-6, H-8, H-2′, H-3′, H-5′ and H-6′ protons suggesting a 4′-oxygenated substitution pattern in ring B; and suggesting an AA′ BB′ spin system. The sugar units in **3** were identified as *β*-d-arabinopyranoside by the analysis of coupling constant of the anomeric signals of sugar proton at *δ* 5.06 (*J* = 7.0 Hz, H-1′′), 4.98 (*J* = 7.2 Hz, H-1′′′). Five broad singlets at *δ* 1.02, 1.11, 1.14, 1.18 and 1.26 all integrated for Me-26′′′′, Me-27′′′′, Me-28′′′′, Me-29′′′′ and Me-30′′′′ and broad signals at *δ* 3.76 and 3.83 were assigned to methoxy protons at C-7 and C-4′. The remaining sugar protons appeared as multiplets at *δ* 4.36–3.57 for H-2′′, H-3′′, H-4′′ and H-2′′′, H-3′′′ and H-4′′′ protons. A one-proton double doublet at *δ* 3.47 (*J* = 5.1, 8.5 Hz) accounted for an oxygenated methine H-3*α* proton. The remaining methylene and methine protons resonated between *δ* 2.86 and 1.37.

The ^13^C NMR spectrum of **3** showed signals for C-4 flavone carbonyl carbon at *δ* 182.18, other flavones carbons at *δ* 162.85 (C-2), 102.63 (C-3), 161.33 (C-5), 101.09 (C-6), 164.50 (C-7), 96.11 (C-8), 158.27 (C-9), and 104.91 (C-10), 128.94 (C-1′), 122.99 (C-2′), 112.51 (C-3′), 154.42 (C-4′), 115.24 (C-5′), 116.54 (C-6′) and anomeric carbons at *δ* 103.12 (C-1′′), 94.51 (C-1′′′), aliphatic chain methyl carbons at *δ* 23.42 (C-26′′′′), 23.81 (C-27′′′′), methoxy carbons at *δ* 56.41 and 56.86, and oxygenated methine carbon at *δ* 77.90 (C-3′′′). The presence of C-2′′ at *δ* 70.70 and C-2′′′′ at *δ* 74.61 supported C_2__→1_ linkages of the suger units.

The ^1^H-^1^H COSY spectrum of **3** showed correlations of H-6 with H-8; H-2′ with H-3′ and H-6′; H-5′ with H-6′; H-2′′ with H-1′′, H-2′′ and H-1′′′; H-2′′′ with H-1′′′; and H_3_-9′′′′ with H-7′′′′ and H-8′′′′. The HMBC spectrum of **3** exhibited interaction of H-1′′ with C-5 and C-2′′; H-1′′′ with C-2′′, confirming (2→1) sugar units and attachment of the innermost sugar at C-5. The arabinopyranosyl sugar was located at the C-5 position of the flavones skeleton according to long-range HMBC correlation between at *δ* 161.33 and the anomeric H-1′′ at *δ* 5.06 as well as at *δ* 6.20. The H-6′′′′ correlated with C-7′′′′; H-30′′′′ with C-14′′′′; H-21′′′′ with C-20′′′′, C-21′′′′ and H-25′′′′ with C-24′′′′, C-26′′′′, C-27′′′′ (Figure 6). The HSQC correlations were used to assign all protons and carbon atoms with the corresponding units and established possible links with other parts of the molecule. Some important correlations in the HSQC spectrum are H-1′′ with C-1′′; H-1′′′ with C-1′′′. The ^1^H and ^13^C NMR values of flavone moieties were compared with those described for similar compounds in the literature. On the basis of the analysis of the spectroscopic data above and 2D NMR (COSY, HSQC and HMBC) the structure of **3** has been formulated as 5-hydroxy-7, 4′-dimethoxyflavone-5-*O*-*β*-d-arabinofuranosyl-(2′′→1′′′)-*O*-*β*-d-arabinopyranosyl-2′′′-*O*-lanost-5-ene, which is a new compound.

Compound **4** was obtained a semi-solid gummy from EtOAc eluants. The IR spectrum displayed absorption bands for hydroxyl groups (3515, 3420, 3356, 3272 cm^−1^), carbonyl groups (1722, 1653 cm^−1^), and benzene ring (1602 cm^−1^). On the basis of the ^13^C NMR and mass spectra, the molecular ion peak of **4** was consistent with the molecular formula of a flavonol diferuloxy feruloyl pentaglycosidic ester *m*/*z* 1361, C_65_H_85_O_31_. High resolution ESI/FTMS provided the exact mass of the protonated molecular ion peak with this molecular formula. The fragmentation pattern of compound **4** is shown in Figure 5.

The ^1^H NMR spectrum of **4** showed nine one-proton doublets and double doublets at *δ* 7.63 (*J* = 2.0 Hz), 7.60 (*J* = 2.0 Hz), 7.41 (*J* = 2.4 Hz), 7.32 (*J* = 7.5 Hz), 7.26 (*J* = 6.5 Hz), 7.13 (*J* = 8.0 Hz), 7.07 (*J* = 2.0, 6.5 Hz0, 7.01 (*J* = 2.0, 7.5 Hz), and 6.82 (*J* = 2.4, 8.0 Hz) which were assigned for H-2, H-2′, H-2′′, H-5, H-5′, H-5′′, and H-6, H-6′, H-6′′ aromatic protons. Six one-proton doublets at 6.56 (*J* = 15.7 Hz), 6.50 (*J* = 15.7 Hz), 6.46 (*J* = 16.3 Hz), 6.43 (*J* = 15.7 Hz), 6.38 (*J* = 15.5 Hz), and 6.23 (*J* = 15.7 Hz) were attributed to vinylic protons for H-7, H-7′, H-7′′, H-8, H-8′, H-8′′. Five one-proton doublets at 6.94 (*J* = 3.5 Hz), 6.80 (*J* = 3.0 Hz), 6.78 (*J* = 5.0 Hz), 6.73 (*J* = 6.0 Hz), and 6.71 (*J* = 3.5 Hz), were attributed to *α*-oriented anomeric H-1, H-1b, H-1c, H-1d, and H-1e protons, respectively. The other sugar protons appeared between *δ* 5.56–3.28. The ^13^C NMR spectrum of **4** showed for free aromatic carbons at *δ* 147.69 (C-2), 137.17 (C-50, 127.68 (C-6), 147.32 (C-2′), 135.02 (C-5′), 124.24 (C-6′), 147.11 (C-2′′), 132.73 (C-5′′), and 122.30 (C-6′′) and oxygenated carbons at *δ* 154.20 (C-3), 149.46 (C-4), 150.99 (C-3′), 149.26 (C-4′), and 150.63 (C-3′′), and 149.01 (C-4′′). The anomeric carbons were appeared at *δ* 107.77 (C-1a), 106.90 (c-1b), 105.11 (C-1c), 104.46 (C-1d), 102.78 (C-1e), and other sugar carbons resonated between at *δ* 79.35–62.61. 

**Figure 7:**
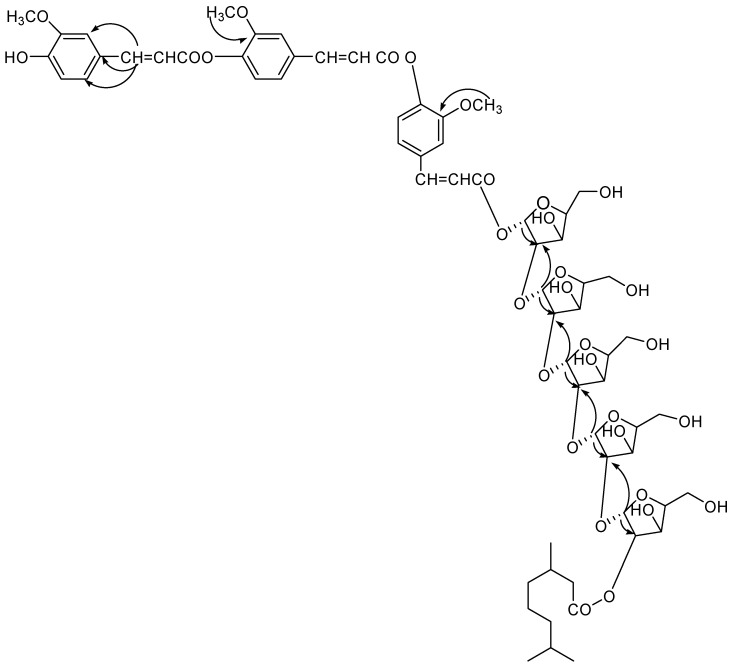
HMBC correlation of compound **4**.

The ^1^H-^1^H COSY spectrum of **4** showed correlations of H-2 with H-5 and H-6; H-2′ with H-5′ and H-6′; H-2′′ with H-5′′ and H-6′′; H-2a with H-1a, H-3a; H-2b with H-1b and H-3b; H-2c with H-1c and H-3c; H-2d with H-1d and H-3d; H-2e with H-1e and H-3e. The HMBC spectrum (Figure 7) of **4** exhibited interactions of H-7 with C-1, C-2 and C-6; CH_3_O with C-3′, C-3′′; H-1a with C-9′′ and C-2a; H-1b with C-2a, C-2b; H-2c with C-1c, C-2b; H-2d with C-1d, C-2c; H-1e with H-2e and 1d. The HSQC correlations were used to assign all protons and carbon atoms with the corresponding units, and established possible links with other parts of the molecule; some important correlations in HSQC spectrum are H-1a with C-1a, H-1b with C-1b, H-1c with C-1a, H-1d with C-1d and H-1e with C-1e.On the basis of spectroscopic data the structure compound **4** assigned as 4′,4′′-diferuloxy feruloyl-*O*-*α*-d-arabinopyranosyl-(2a→1b)-*O*-*α*-d-arabinopyranosyl-(2b→1c)-*O*-*α*-d-arabinopyranosyl-(2c→1d)-*O*-*α*-d-arabinopyranosyl-(2d→1e)-*O*-*α*-d-arabinopyranosyl-2e-3′′′, 7′′′-dimethylnonan-1′′′-oate.

### 2.1. Cytotoxicity of Seven Chemical Constituents against Raw 264.7 Cells

**Figure 8:**
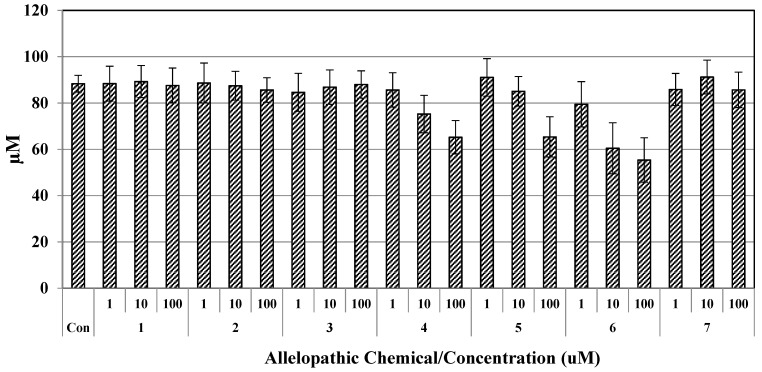
Cell viability of RAW 264.7 cell exposed to isolated chemical constituents of the straw and leaves of *O. sativa*. Solutions of the seven allelopathic chemical constituents (10% MeOH in distilled water); Data represent the absorbance values obtained from the MMT assay over 24 h. The cell viability percentage was the mean absorbance of seven allelopathic chemical constituents at different concentrations (1, 10, 100 μM) divided by that of the corresponding control group. The bars represent the mean ± SD obtained in three independent experiments.

The cytotoxicity of allelopathic chemical constituents towards RAW 264.7 cells was determined by MTT assay. Results are shown in Figure 8. The data indicate that the rate of cell viability decreased significantly (*p* < 0.05) with increasing concentrations of compound (**4**), (**5**), and (**6**) compared to those of the control group, and cytotoxicity increased dose dependently. In the current study, the absorbance of each experiment was similar among the three parallel experiments. Overall results showed that the inhibitory effect of compounds (**4**), (**5**), and (**6**) at 1 μM were similar with slight differences. According to the results, 10% MeOH in distilled water displayed a higher cytotoxic effect (88.36%) than water (100%), while for 100 μM of compound (**4**), (**5**), and (**6**) in 10% MeOH, the corresponding cell viability observed in RAW 264.7 cells were 65.24%, 65.37%, and 55.39%, respectively. Compounds **1**, **2**, **3** and **7** at 100 μM had little or no toxicity, whereas compound **4**, **5**, and **6** inhibited the growth of cells at higher concentrations, the viability rate of all chemical constitutes did not exceed 65.0% at doses up to 100 μM, respectively (Figure 8). Taher et al. (2016) reported [27] that the biflavonoid, morelloflavone, isolated from *Garcinia prainiana* showed no obvious inhibition of RAW 264.7 macrophage cell viability [27]. Also, the cytotoxicity of the novel synthesized flavone derivatives was investigated in terms of the structure–anti-inflammatory activity relationship. Flavone derivatives possessing a 5,7-dimethoxy or 3′,4′-dihydroxy moiety were less toxic than the other compounds. From the above results, we suggest that compounds (**1**), (**2**), (**3**), and (**7**) might be useful, safe, and have biological potential.

Our data showed that some of the flavone derivatives exerted different dose-dependent effects among the seven isolated compounds from straw and leaves of rice against germination and shoot and root growth of barnyardgrass (*E. oryzicola*), a noxious weed in rice fields.

Compound **1** showed inhibitory activity in the form of decreased MGT of barnyardgrass at all concentrations, and at the highest dose, GS and CVG were inhibited. While compound **2** decreased the FGP and GS, compound **3** decreased the FGP and MGT of the same seed. The germination index of tested seeds was not affected by the compounds tested, except for the flavone compounds, as shown in Table 5. In fact, only three compounds (**1**–**3**) inhibited the germination of barnyardgrass.

**Figure 9:**
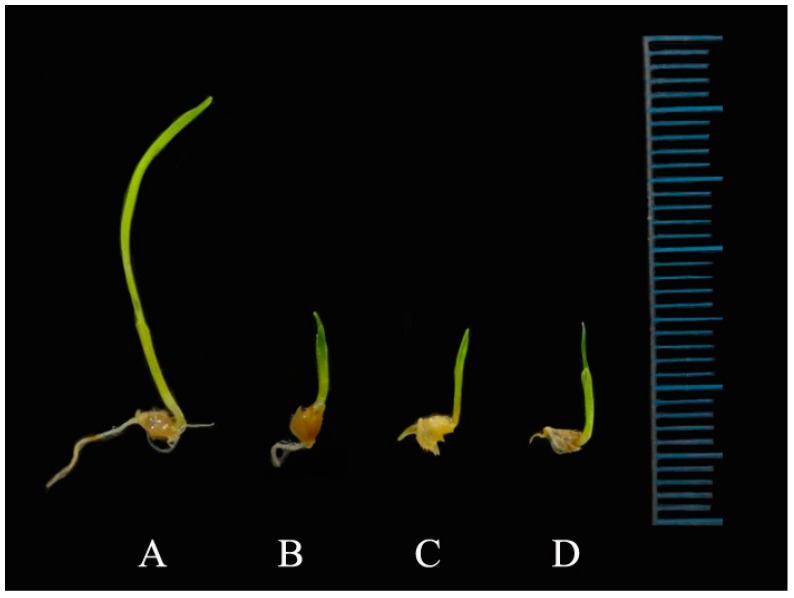
Morphological characterization of seed germination of *E. oryzicola* according to treatment of isolated compounds from rice (*O. sativa*) after 14 days. (**A**) DW+ 10% MeOH, (**B**) compound **1** 500 µM, (**C**) compound **2** 500 µM, (**D**) compound **3** 500 µM.

Data concerning the activity of the tested flavones against the shoot and root elongation of barnyardgrass are reported in Table 6. Compounds **1**–**3** showed inhibitory activity in the shoot and root elongation of *E. oryzicola*, depending on the concentration: at the lowest dose (100 µM), it was not affected, but at the highest dose it was inhibitory. Also, treatment with the same compound decreased the fresh and dry weight of *E. oryzicola* seedlings (Table 7, Figure 9). The flavone compounds showed the greatest inhibitory activity against radical elongation: it is possible that the entry of water through the integument during the germination process produced the entry of bioactive compounds, by mass flow, which began their physiological activities in the next phase of root growth [28].

Among the seven isolated chemicals, it is observed that the most active inhibitory compounds are flavones, characterized by a 4-oxo function, the 2, 3 double bonds in the C-ring, and one or more OH groups. De Martino et al. (2012) reported [29] that substituted compounds are more active against radical elongation than unsubstituted flavonoids, and the most active flavones are generally methoxy-substituted. Comparing the tested flavones, the absence of a 3-substituted residue in the ring C and of a 6-OH group in the ring A (compound **3**) appears unimportant for the allelopathic activities. In addition, the combination of 4-carbonyl function and C-2 to C-3 double bond appears to be very important for the appearance of allelopathic activities. The flavone derivatives with catechol structures showed significant promoting activity towards barnyardgrass seeds and they present the characteristic of an easy rotation of the B-ring with respect to the A- and C-ring systems and thus have structural flexibility [30]. Our results led us to hypothesize that the catechol orientation of the B-ring may be responsible for the allelopathic effects.

In terms of antioxidant activity, DPPH tests detected **1** > **4** > **2** > **5** > **3** as the most active compounds among the seven isolated chemicals (Table 8). Compound **1** showed the greatest activity, possibly due to the 2,3-double bond in conjugation with a 4-oxo function responsible for electron dislocation from the B-ring and the presence of both 5- and 7-hydroxyl groups. Also, compound **1** had higher DPPH radical scavenging activity due to presence of free hydroxyl groups and fewer methoxy group substituents than compounds **2** and **3**. Glycosylation of flavonoids diminishes their activity when compared to the corresponding aglycones and suppression of antioxidant activity by *O*-methylation [31,32]. Although the ratio of methoxy to hydroxyl substituents does not necessarily predict the scavenging ability of a flavonoid, the B-ring is particularly sensitive to the position of the methoxy group.

Compound **2** has more effective free radical scavengers than compound **3,** which may be ascribed to its greater number of hydroxyl groups. The presence of certain hydroxyl groups on the flavonoid rings enhances antioxidant activity [29]. Our data showed no relationship between the allelopathic effect and the antioxidant properties of the **7** isolated chemicals. The mechanisms of flavonoid action are less understood than those of phenolic acids. Einhellig (2004) states that the flavonoids are the second most active class of allelochemicals inhibiting mitochondrial oxygen uptake [33]. Moreland and Novitsky (1987) concluded that flavonoids act primarily as electron transport inhibitors through perturbation of the mitochondrial inner membrane [34]. Some allelopathic flavonoids are potent inhibitors of energy metabolism, blocking mitochondrial and chloroplast functions [33], by which they can influence cell growth, ATP production, and auxin function. Despite flavonoids having multiple targets of plant physiology, the allelopathic effect of numerous flavonoids is mediated, in part, by the fact that in almost all situations, a number of different compounds are acting at the same time. In our results, compounds **1**–**3**, showed an allelopathic effect that inhibited seed germination and seedling growth in barnyardgrass by affecting other germination factors. The results indicate that the allelopathic effects of the isolated chemicals may be induced by a combination of compounds. Also, flavones are unlikely to be the major allelochemicals isolated form rice since their concentrations in paddy fields never reach toxic levels, but it is suggested that flavones might be one component in a mixture of chemicals that, when present simultaneously, have allelopathic effects. The significance of allelochemicals is gaining more and more attention in agriculture because their interactions could be employed for reducing weed growth.

## 3. Materials and Methods

### 3.1. Chemical and Instruments 

Melting points of the compounds were determined using a model IA9100 melting point apparatus (Electrochemical Engineering, Seoul, Korea). Optical rotations were measured with a model AA-10 polarimeter (Instrument Ltd., Seoul, Korea). Ultraviolet (UV) spectra were measured with a TU-1800_PC_ UV-vis spectrophotometer (Instrument Ltd., Seoul, Korea). Infrared (IR) spectra were recorded after compound mixing with potassium bromide (KBr) on a Thermo Scientific FT-IR model Nicolet 6700 (USA) (Waltham, MA, USA) spectrophotometer at the Korea Institute of Science and Technology (KIST) Seoul, Korea. Both nuclear magnetic resonance (NMR) spectra ^1^H (600 MHz) and ^13^C NMR (125 MHz) were measured with a Bruker Avance-600 spectrometer (Billerica, MA, USA) sing deuterated solvents, and the machine was available at National Instrumentation Centre for Environmental Management (NICEM), College of Agriculture and Life Science, Seoul National University (SNU), Seoul, Korea. NMR spectra were recorded in deuterated chloroform, pyridine-*d*_5_, and methanol-*d*_4_ using tetramethylsilane (TMS) as an internal standard, with chemical shifts expressed in parts per million (*δ*) and coupling constants (*J*) in Hertz. High-resolution electrospray ionization Fourier transform (ESI/FT) mass spectra were recorded on a Thermo-Finnigan LTQ-Orbitrap instrument (Thermo Scientific, Bartlesville, OK, USA) equipped with Dionex U 3000 HPLC system with UV-VIS detector (SPD-10A). A C18 ODS column was used with a mobile phase of 0.1% TFA in acetonitrile: water (80:20) and a flow rate of 4 mL^−1^. The National Instrumentation Centre for Environmental Management (NICEM), Seoul National University). All chemicals were of analytical grade. *n*-Hexane, ethyl acetate, methanol, ethanol, sulfuric acid (H_2_SO_4_), and vanillin were purchased from Daejung Chemicals and Metals (Seoul, Korea). Thin layer chromatography (TLC) was performed on precoated silica gel 60 F_254_ plates (Merck, Darmstadt, Germany). Visualization of TLC plates was performed in a developing glass chamber and, after drying, they were dipped in solution of using a 5% vanillin and H_2_SO_4_ in ethanol spray reagent (5:5:90). Column chromatography was performed using silica gel (70–230 mesh) and LiChroprep RP-18 (40–63 μm; Octadecyl silica (ODS) gel) from Merck. Standards were purchased from Sigma-Aldrich (St. Louis, MO, USA).

### 3.2. Plant Materials

The rice plant (*O. sativa*, leaves and straw) used in the present study was collected after the harvesting of rice cereal at Konkuk University experimental farm Yeoju, Korea in October 2013. The collected samples were dried in the laboratory at a temperature range of 30–35 °C for 3 weeks, with some modifications to the procedure performed in a previous study. A voucher specimen (reference code ILPUM variety) has been dried and deposited in the herbarium of the Department of Applied Life Science, Konkuk University.

### 3.3. Extraction of Rice Plant 

Dried rice plant (2.4 kg, leaves and straw, powdered) was immersed in methanol (10 L × 3) for 1 week at room temperature 25–30 °C. Then, the supernatant was concentrated under vacuum to yield 190 g extract. This freeze-dried extract was again dissolved in methanol for removing fat and kept in a refrigerator for 3 h. The fat was crystallized and filtered through a sintered funnel. The filtrate was then concentrated to obtain 132 g extract.

### 3.4. Isolation and Identification of Compounds from Methanol Extract

The methanol extract (132 g) was subjected to normal-phase column chromatography (CC) on silica gel (70–230 mesh, 1.2 kg, 1500 × 45 mm), yielding 50 fractions (each fraction 500 mL) with the following eluents: fraction 1–5 in hexane, fractions 6–10 in hexane:ethyl acetate (EtOAc) (8:2), fractions 11–15 in hexane:EtOAc (6:4), fractions 16–20 in hexane:EtOAc (4:6), fractions 21–25 in hexane:EtOAc (8:2), fractions 26–30 in EtOAc, fractions 31–35 in EtOAc:MeOH (9.5:0.5), and fractions 36–40 in EtOAc:MeOH (9:1), fractions 41–45 in EtOAc:MeOH (8.5:1.5), and fractions 45–50 in EtOAc:MeOH (8:2). Fifteen fractions were collected from fractions 26–30 after rechromatography on silica gel with chloroform and methanol. Fractions 14 and 15 were subjected to CC over LiChroprep RP-18 (ODS column) and eluted sequentially with methanol containing 80, 60, 40 20, 10, and 0% water to yield three new compounds: **1** (42 mg), **2** (59 mg), and **3** (49 mg). Fractions 36–40 were combined after chromatography on a silica gel column with chloroform and methanol, rechromatographed over LiChroprep RP-18, eluted sequentially with methanol containing 80, 60, 40 20, 10, and 0% water to yield **4** (49 mg), and **5** (69 mg), **6** (61 mg), and **7** (56 mg).

*5, 7-Dihydroxy-4′-Methoxyflavonol-3-O-β-d-Arabinopyranosyl-(2′′→1′′′)-O-β-d-Arabinopyrnosyl-2′′′-O-3′′′′, 7′′′′-Dimethylnonan-1′′′′-Oate* (**1**): Yellow powder; mp. 223–225 °C; R_f_ 0.59 (chloroform:methanol:9:1); [*α*]_D_^24^ –39.1 (*c*, 0.7, MeOH); IR (KBr) ν_max_ 3450, 3358, 3271, 2939, 2836, 1721, 1685, 1602, 1514, 1428, 1367, 1226, 1164, 1121, 1025, 833, 752 cm^−1^; for ^1^H and ^13^C NMR spectroscopic data, see Table 1; ESIMS *m*/*z* (rel. int.): 719 [M + H]^+^ (C_36_H_47_O_15_) (1.3), 299 (10.3), 287 (37.1), 155 (97.2); HRFTMS *m*/*z* 719.2915 (calcd for C_36_H_47_O_15_, 719.2921).

*5-Hydroxy-7, 4′-Dimethoxyflavone-5-O-α-d-Arabinopyranosyl-(2"→1′′′)-O-α-d-Arabinopyranosyl-2′′′-3′′′′, 7′′′′-Dimethylnonan-1′′′′-Oate* (**2**): Yellow powder; mp. 220–225 °C; R_f_ 0.49 (chloroform: methanol:9:1); [*α*]_D_^24^ −69.1 (*c*, 0.9, MeOH); IR (KBr) ν_max_ 3451, 3369, 3283, 2938, 2832, 1720, 1607, 1513, 1456, 1424, 1346, 1260, 1174, 1118, 1024, 836 cm^−1^; for ^1^H and ^13^C NMR spectroscopic data, see Table 2; ESI MS *m*/*z* (rel. int.): 717 [M + H]^+^ (C_37_H_49_O_15_) (2.4), 429 (5.1), 419 (5.3), 297 (6.8), 287 (4.7), 155 (100); HRFTMS *m*/*z* 717.3123 (calcd for C_37_H_49_O_14_, 717.3128).

*5-Hydroxy-7, 4′-Dimethoxyflavone-5-O-β-d-Arabinofuranosyl-(2"→1′′′)-O-β-d-Arabinopyranosyl-2′′′-O-Lanost-5-Ene* (**3**): Semi-solid; R_f_ 0.69 (chloroform:methanol:9:1); [*α*]_D_^24^ –59.1 (*c*, 0.8, MeOH); IR (KBr) ν_max_ 3450, 3355, 3281, 2937, 2841, 1690, 1635, 1525, 1428, 1367, 1228, 1164, 1120, 1026, 835 cm^−1^; for ^1^H and ^13^C NMR spectroscopic data, see Table 3; ESIMS *m*/*z* (rel. int.): 972 [M + H]+ (C_57_H_81_O_13_) (2.2), 675 (4.1), 430 (8.3), 427 (20.5), 297 (15.7); HRFTMS *m*/*z* 973.5677 (calcd. for C_57_H_81_O_13_, 973.5682).

*4′, 4′′-Diferuloxy Feruloyl-O-α-d-Arabinopyranosyl-(2a→1b)-O-α-d-Arabinopyranosyl-(2b→1c)-O-α-d-Arabinopyranosyl-(2c→1d)-O-α-d-Arabinopyranosyl-(2d→1e)-O-α-d-Arabinopyranosyl-2e-3′′′, 7′′′-Dimethylnonan-1′′′-Oate* (**4**): Gummy; R_f_ 0.79 (chloroform:methanol:9:1); [*α*]_D_^24^ −79.1 (*c*, 0.9, MeOH); IR (KBr) ν_max_ 3515, 3420, 3356, 3237, 2916, 2848, 1722, 1653, 1602, 1513, 1480, 1425, 1336, 1264, 1223, 1116, 1070, 1022, 718 cm^−1^; ESIMS *m*/*z* (rel. int.): 1361 [M + H]^+^ (C_65_H_85_O_31_) (1.3), 1205 (1.6), 1189 (1.8), 544 (13.8), 528 (4.7), 368 (5.2), 352 (5.5), 303 (9.1), 289 (9.7), 192 (6.4), 176 (4.9), 155 (19.3); HRFTMS *m*/*z* 1361.5076 (calcd for C_65_H_85_O_31_, 1361.5081)_._

### 3.5. Acid Hydrolysis of ***1**−**4***

Compounds **1**−**4** (10 mg each) were heated at 70−80 °C with diluted 2 M hydrochloric acid (2.5 mL) in 70% aqueous ethanol (3 mL) for 30 min. The reaction mixture was dried under vacuum, neutralized with aqueous sodium bicarbonate, and extracted with chloroform (3 × 5 mL) to separate the flavonoid moiety. The chloroform extract was washed with water (3 × 5 mL), dried over anhydrous sodium sulfate, and evaporated to produce aglycone moieties. The mother liquor, after separation of the flavonoid, was treated with dilute 2 M hydrochloric acid to liberate the other aglycone moiety and was re-extracted with chloroform (3 × 5 mL) to obtain the compound. The presence of sugars in the aqueous solution was determined by co-TLC.

### 3.6. DPPH Radical Scavenging Activity

The DPPH radical scavenging activity of the seven of chemical constituents isolated from rice plant (straw and leaves) of *O. sativa* was determined by the method of Gyamfi et al. [19]. Initially, 400 µL of sample at different concentrations (0, 200, 400, 600, 800, 1000 µM) was mixed with 1.6 mL of 100 mM Tris-HCl buffer (pH 7.4) and 2 mL of 0.5 mM DPPH (Sigma, St. Louis, MO, USA) dissolved in methanol. The reaction mixture was incubated for 20 min at room temperature. The control contained all reagents without the sample, and 10% methanol was used as the blank. Measurements were performed in triplicate. DPPH radical scavenging activity was determined by measuring absorbance at 517 nm using a spectrophotometer (UV-1650PC UV/VIS spectrophotometer (Shimadzu, Kyoto, Japan). The IC_50_ value (µM) is the concentration at which scavenging activity is 50%.

### 3.7. Cell Culture 

Raw 264.7 cells were purchased from American Type Culture Collection (ATCC, Manassas, VA, USA) and grown in Dulbecco′s modified eagle medium (DMEM) supplemented with 10% fetal bovine serum (FBS) and 1% penicillin/streptomycin. All the cells were maintained at 37 °C in a humidified atmosphere of 5% CO_2_ throughout the study and routinely grown in 25 cm^2^ culture flasks and trypsinized to harvest after attaining confluence. Meanwhile, some differences were considered regarding trypsinization procedure for RAW 264.7 semi adherent cells versus two other adherent cells. Following centrifugation (1300 *g* for 7 min), cells were resuspended in the culture medium and used for the following study.

### 3.8. Cytotoxicity Test-MTT Assay

To evaluate the cytotoxicity of the seven isolated chemicals on RAW 264.7 cell, viability tests were applied using the MTT colorimetric assay. Briefly, all cell lines were seeded in 96-well plates at a density of 2 × 10^5^ cells per well and then incubated at 37 °C in 5% CO_2_ to allow cell attachment. The medium was removed and replaced with fresh medium containing various concentrations (0, 10, and 1000 μM) of seven of chemical constituents. After treatment for 24 h, 100 μL MTT (1 mg/mL) was added to each well and the plate was further incubated. Four hours later, all remaining supernatant was removed and 100 μL of DMSO was added to each well to dissolve the resulting formazan crystals. Finally, absorbance was read at 570 nm using a multilabel plate reader (VICTOR^TM^X3, Perkin Elmer, Waltham, MA, USA) and the cell viability percentage was calculated using the equation: (mean OD of treated cells/mean OD of control cells) × 100.

### 3.9. Seeds Germination and Growth Records 

Seeds of barnyardgrass (*E. oryzicola*) found in rice paddies of Korea were collected during 1997 and 1998. The seeds were dried (water content 12.04% at fresh weight basis) and stored at 5 °C in the dark. Before the start of experiment, the seeds were soaked with distilled water for one day to remove the germination inhibition compound in the seed coat (WP, water priming), and their germination was determined, which was >80% in all cases. The germination test was carried out in sterile petri dishes (15 cm) lined with Whatman No. 2 filter paper (Whatman Co., Maidstone, UK). Seeds of maize were sterilized with NaOCl 10% for 2–3 min. Thereafter, the seeds were thoroughly rinsed three times with sterile water. Aqueous extracts of different concentrations (10 mL) were pipetted to the filter paper placed in petri dishes and distilled water was used as control treatment. The petri dishes were set in the laboratory at room temperature ranging from 28–30 °C. The experiment extended over a period of 7 d to allow the last seed’s germination. A seed was considered as germinated when a radical emerged. Germination was recorded on daily basis. The results were determined by counting the number of germinated seeds and measuring the lengths of both root and shoot. At the end of the last day of germination, germination characteristics and seedling growth including fresh and dry weight were recorded. Final germination percentage (FGP) was calculated by using FGP = Ng/Nt × 100, where Ng is total number of germinated seeds and Nt is total number of evaluated seeds. The germination index (GI) of the seeds were estimated as follow GI = (13 × N1) + (12 × N2) + ... + (1 × N13). N1, N2 and … are the number of germinated seeds at first day, second, and other days and numbers 9, 10 and ... are respectively the weights imposed on the number of seeds germinated at first, second, and other days. Also, the coefficient of velocity of germination (CVG) was calculated by using CVG = 100 × ΣNi/ΣNiTi formula, where Ni is number of germinated seeds per day and Ti is number of days from the start of the experiment. Germination speed (Rs) was estimated based on the Magour method and by using the following equation, Rs = ΣSi/Di ^20^, where Si is the number of germinated seeds in it h day and Di is the day number. The calculation of mean of germination time (MGT) was done using the following equations: MGT = ΣNiTi/ΣNi = 100/CVG [21], where Ni is number of germinated seeds for each day and Ti is number of days as of the start of experiment. 

## 4. Statistical Analysis

The percent seed germination inhibition values were determined and subjected to analysis of variance (ANOVA). Critical differences (CDs) were calculated at *p* < 0.05.

## 5. Conclusions

We have reported the isolation of four new and three known compounds from *O. sativa*, along with their biological activities, including cytotoxicity and allelopathic activities, on weed germination. To the best of our knowledge, there has been no prior report on the phytochemistry of *O. sativa* straw and leaves.

## Figures and Tables

**Table 1:** ^1^H NMR and ^13^C NMR Spectroscopic data of compound **1**.

Position	^1^H NMR	^13^C NMR
1	-	-
2	-	149.36
3	-	131.27
4	-	175.32
5	-	152.14
6	6.25 s	98.25
7	-	161.01
8	6.31 s	94.35
9	-	149.32
10	-	106.52
1′	-	121.76
2′	7.65 dd (7.5,1.5)	129.24
3′	6.81 dd (7.5, 2.0)	116.69
4′	-	146.29
5′	6.78 dd (2.0,8.5)	111.72
6′	7.43 d (8.5)	127.74
1′′	5.43 d (7.1)	104.89
2′′	4.18 m	89.12
3′′	3.88 m	77.88
4′′	3.83 m	73.81
5′′	3.30, 3.29 d (3.0, 3.0)	64.41
1′′′	4.96 d (7.2)	93.96
2′′′	4.32 m	79.53
3′′′	3.86 m	75.14
4′′′	3.80 m	71.75
5′′′	3.59 d (11.5)	63.62
1′′′′	-	171.24
2′′′′	2.07, 2.03 dd (9.0, 8.5)	46.13
3′′′′	1.89 m	45.05
4′′′′	1.32 m	35.06
5′′′′	1.18 m	30.16
6′′′′	1.13 m	29.30
7′′′′	1.58 m	44.54
8′′′′	0.96 d (7)	20.60
9′′′′	1.01 d (8)	20.83
10′′′′	1.28 d (6.5)	28.75
OMe	3.36, 3.86 br s	56.41

**Table 2:** ^1^H NMR and ^13^C NMR Spectroscopic data of compound **2.**

Position	^1^H NMR	^13^C NMR
1	-	-
2	-	162.78
3	3.68 s	106.96
4	-	183.94
5	-	158.85
6	6.43 s	101.70
7	-	164.75
8	6.64 s	96.32
9	-	154.79
10	-	104.78
1′	-	120.85
2′	7.15 d (8.5)	127.70
3′	6.81 d (8.5)	115.86
4′	-	148.74
5′	6.77 d (10.5)	111.80
6′	7.13 d (10.5)	115.69
1′′	5.01 d (6.5 )	105.22
2′′	3.88 m	78.51
3′′	3.80 m	77.88
4′′	3.68 m	74.26
5′′	3.53 d (9.0)	62.61
1′′′	4.93 dd (5.0)	96.32
2′′′	4.44 dd (5.0, 5.5)	87.55
3′′′	3.72 m	74.46
4′′′	3.65 m	71.31
5′′′	3.41 d (9.5 )	61.96
1′′′′	-	171.26
2′′′′	2.26 dd (8.0,7.5)	49.81
3′′′′	1.89 m	44.62
4′′′′	1.31 m	34.99
5′′′′	1.29 m	30.73
6′′′′	1.27 m	30.48
7′′′′	1.58 m	38.33
8′′′′	0.98 d (6.6)	26.45
9′′′′	0.93 d (6.4)	26.07
10′′′′	1.16 d (6.5)	30.14
7, 4′ (OMe)	3.29, 3.34, each br s	56.83, 57.20

**Table 3:** ^1^H NMR and ^13^C NMR Spectroscopic data of compound **3**.

Position	^1^H NMR	^13^C NMR
1	-	
2	-	162.83
3	6.54 s	102.63
4	-	182.18
5	-	161.33
6	6.20 d (2.0)	101.09
7	-	164.50
8	6.31 d (2.0)	96.11
9	-	158.27
10	-	104.91
1′	-	128.94
2′	7.41 dd (9.0, 3.5)	122.99
3′	6.75 dd (9.0, 2.0)	112.51
4′	-	154.42
5′	6.72 dd (8.5, 2.0)	115.24
6′	7.05 dd (8.5, 3.0)	116.54
1′′	5.06 d	103.12
2′′	4.36 m	70.70
3′′	4.01 m	74.80
4′′	3.60 m	71.35
5′′	3.36 d (5.6)	62.86
1′′′	4.98 d (7.2)	94.51
2′′′	4.16 m	74.61
3′′′	3.86 m	74.13
4′′′	3.57 m	71.37
5′′′	3.31 d (3.0)	62.54
1′′′′	-	32.59
2′′′′	1.22 d (6.5)	26.72
3′′′′	3.47 dd (5.1, 8.5)	77.90
4′′′′	-	42.38
5′′′′	-	145.94
6′′′′	5.44 dd (4.0, 1.5)	121.92
7′′′′	-	29.75
8′′′′	-	41.57
9′′′′	-	54.01
10′′′′	-	38.63
11′′′′	-	20.97
12′′′′	-	30.09
13′′′′	-	44.65
14′′′′	-	51.34
15′′′′	-	33.72
16′′′′	-	35.93
17′′′′	-	49.51
18′′′′	0.95 br s	20.21
19′′′′	1.28 brs	20.25
20′′′′	-	37.75
21′′′′	-	21.18
22′′′′	-	30.73
23′′′′	-	25.14
24′′′′	-	45.43
25′′′′	-	25.14
26′′′′	1.02 d (6.6)	23.42
27′′′′	1.11 d (7.5)	23.81
28′′′′	1.14 brs	23.81
29′′′′	1.18 brs	30.73
30′′′′	1.26 brs	24.63
OMe	3.83 brs	56.41
OMe	3.76 brs	56.86
10 × CH, 5 × CH·(25H·)	2.86–1.37	-

**Table 4:** ^1^H NMR and ^13^C NMR Spectroscopic data of compound **4**.

Position	^1^H NMR	^13^C NMR
1	-	145.52
1a	6.94 d (3.5)	07.77
1b	6.80 d (3.0)	106.90
1c	6.78 d (5.0)	105.11
1d	6.73 d (6.0)	104.46
1e	6.71 d (3.5)	102.78
2	7.63 d (2.0)	147.69
2a	4.38 m	78.29
2b	4.35 m	78.04
2c	4.27 m	75.75
2d	4.24 m	75.16
2e	4.18 m	74.64
3	-	154.20
3a	3.86 m	73.18
3b	3.83 m	72.06
3c	3.81 m	71.70
3d	3.48 m	71.56
3e	3.40 m	71.35
4	-	149.46
4a	5.56 m	93.27
4b	5.41 m	88.78
4c	5.01 m	84.02
4d	4.86 m	83.87
4e	4.52 m	79.35
5	7.32 d (7.5)	137.17
5a	3.37 d (8.5)	63.84
5b	3.33 d (10.0)	64.16
5c	3.30 d (11.5)	64.96
5d	3.28 d (6.5)	65.08
5e	3.26 d (8.1)	62.61
6	7.01 dd (2.0,7.5)	127.68
7	6.56 d (15.7)	119.43
8	6.43 d (15.7)	114.32
9	-	169.87
1′′	-	139.09
2′	7.60 d (2.0)	147.32
3′	-	150.99
4′	-	149.26
5′	7.26 d (6.5)	135.02
6′	7.07 dd (6.5, 2.0)	124.24
7′	6.50 d (15.5)	118.13
8′	6.38 d (15.5)	112.25
9′	-	169.16
1′′	-	138.08
2′′	7.41 d (2.4)	147.11
3′′	-	150.63
4′′	-	149.01
5′′	7.13 d (8.0)	132.73
6′′	6.82 dd (2.4, 8.0)	122.30
7′′	6.46 d (16.3)	116.46
8′′	6.23 d (15.7)	111.28
9′′	-	169.06
1′′′	-	172.38
2′′′	2.65 d (8.0)	53.98
3′′′	2.35 m	34.40
4′′′	1.37 m	34.40
5′′′	1.17 m	33.39
6′′′	1.10 m	32.67
7′′′	1.70 m	35.37
8′′′	1.02 d (6.5)	23.45
9′′′	1.00 d (6.5)	22.12
10′′′	1.26 d (6.0)	26.51
OMe	3.78 br s	57.07
OMe	3.32 br s	56.53
OMe	3.23 br s	54.10

**Table 5:** Germination characteristics of *E. oryzicola* according to treatment of different concentrations of isolated compounds from rice (*O. sativa*) after 7 days.

Sample	Conc. (µM)	FGP ^(1)^ (%)	MGT ^(2)^ (Days)	Gs ^(3)^	CVG ^(4)^	GI ^(5)^
DW		95.33 ± 3.06	6.98 ± 0.09	34.06 ± 1.80	14.32 ± 0.18	6.66 ± 0.26
Con		87.83 ± 8.58	6.62 ± 0.37	29.69 ± 0.96	15.83 ± 0.84	5.84 ± 0.90
1	100	75.00 ± 8.26	6.07 ± 0.26 *	34.04 ± 4.75	16.50 ± 0.68	4.57 ± 0.69 *
	250	85.79 ± 2.89	6.03 ± 0.06 *	31.06 ± 2.29	16.58 ± 0.16	5.17 ± 0.14 *
	500	85.07 ± 1.84	5.92 ± 0.14 *	27.19 ± 1.67 *	16.88 ± 0.41 *	5.04 ± 0.10 *
2	100	78.17 ± 4.93	6.19 ± 0.09	26.47 ± 2.09 **	16.17 ± 0.24	4.83 ± 0.27 *
	250	67.33 ± 6.53 *	6.15 ± 0.38	24.66 ± 0.60 **	16.34 ± 1.45	4.22 ± 0.20 *
	500	68.63 ± 1.27 **	5.79 ± 0.46 *	25.72 ± 4.81 **	17.30 ± 0.97 *	3.92 ± 0.67 **
3	100	75.59 ± 5.45 *	6.23 ± 0.17 *	29.99 ± 2.22	16.06 ± 0.43	4.41 ± 0.79 *
	250	70.57 ± 9.93 *	5.92 ± 0.12 *	28.66 ± 2.16	16.89 ± 0.34	4.43 ± 0.44 *
	500	64.86 ± 2.02 **	5.86 ± 0.16 *	27.01 ± 0.94 *	17.08 ± 0.47 *	3.84 ± 0.36 **
4	100	82.67 ± 6.01	6.76 ± 0.22	26.66 ± 2.83 *	14.81 ± 0.49	5.58 ± 0.17
	250	78.33 ± 8.14	6.96 ± 0.20	24.18 ± 2.91 **	14.99 ± 0.40	5.46 ± 0.64
	500	73.95 ± 3.07 *	6.85 ± 0.22	25.27 ± 1.00 **	14.61 ± 0.47	5.06 ± 0.07
5	100	75.25 ± 5.63	6.16 ± 0.20	31.81 ± 0.78	16.25 ± 0.53	4.94 ± 0.28
	250	77.21 ± 9.85	6.30 ± 0.09	32.16 ± 1.89	15.88 ± 0.22	5.22 ± 0.14
	500	80.12 ± 2.78	6.35 ± 0.30	26.25 ± 1.70 *	15.78 ± 0.76	4.97 ± 0.52
6	100	76.50 ± 3.50	7.17 ± 0.18	29.44 ± 1.67	15.96 ± 0.35	5.48 ± 0.31
	250	77.21 ± 9.32	6.23 ± 0.20	29.39 ± 4.94	15.54 ± 0.81	4.94 ± 0.16
	500	80.12 ± 2.78	5.72 ± 0.27 *	32.35 ± 0.86	16.08 ± 0.52	4.92 ± 0.87
7	100	88.17 ± 3.21	6.81 ± 0.23	28.89 ± 0.07	14.72 ± 0.50	6.01 ± 0.42
	250	85.17 ± 6.01	6.83 ± 0.67	28.21 ± 2.47	14.77 ± 1.52	5.84 ± 0.91
	500	82.17 ± 5.06	6.51 ± 0.30	28.10 ± 1.05	15.34 ± 0.72	5.36 ± 0.56
PBC ^(6)^	100	81.33 ± 1.76	6.66 ± 0.30	28.08 ± 0.26	15.02 ± 0.68	5.42 ± 0.36
	250	78.33 ± 5.13 *	6.33 ± 0.19	28.22 ± 0.76	15.80 ± 0.47	4.96 ± 0.43
	500	77.17 ± 3.79 *	6.69 ± 0.13	25.47 ± 0.71 *	14.97 ± 0.29	5.16 ± 0.30

Mean values ± SD from triplicate separated experiments are shown. * Significantly different from DW+ 10% MeOH (Con) based on the DMRT (*p* < 0.05). FGP ^(1)^: Final germination percentage; MGT ^(2)^: Mean germination time, Gs ^(3)^: Germination speed, CVG ^(4)^: Coefficient of velocity of germination, GI ^(5)^: Germination index, PBC ^(6)^: Prybuticarb.

**Table 6:** Effect of isolated compounds from rice (*O. sativa*) on shoot and root length of *E. oryzicola* after 7 days.

Sample Codes	Seedling Length (cm)/Concentrations (µM)			Root Length (cm)/Concentrations (µM)		
	100	250	500	100	250	500
1	0.92 ± 0.08	0.81 ± 0.08 *	0.71 ± 0.10 *	0.35 ± 0.14	0.21 ± 0.04 *	0.20 ± 0.06 *
2	0.99 ± 0.24	0.87 ± 0.15 *	0.79 ± 0.12 *	0.36 ± 0.19	0.27 ± 0.09 *	0.24 ± 0.09 *
3	0.89 ± 0.14	0.74 ± 0.26 *	0.81 ± 0.12 *	0.31 ± 0.11	0.21 ± 0.15 *	0.22 ± 0.08 *
4	0.89 ± 0.30	1.00 ± 0.20	0.91 ± 0.37	0.32 ± 0.16	0.38 ± 0.15	0.31 ± 0.16
5	1.23 ± 0.39	1.03 ± 0.31	1.28 ± 0.20	0.42 ± 0.21	0.41 ± 0.12	0.42 ± 0.10
6	0.97 ± 0.14	1.40 ± 0.14	1.46 ± 0.58	0.37 ± 0.11	0.48 ± 0.19	0.48 ± 0.38
7	1.15 ± 0.16	1.21 ± 0.33	1.04 ± 0.21	0.36 ± 0.14	0.36 ± 0.16	0.38 ± 0.14
Pyributicarb	0.84 ± 0.07 *	0.99 ± 0.09 *	1.00 ± 0.06	0.24 ± 0.04 *	0.25 ± 0.04 *	0.25 ± 0.05 *
DW+ 10% MeOH	1.17 ± 0.36			0.33 ± 0.27		
DW	4.57 ± 0.43			2.04 ± 0.13		

Mean values ± SD from triplicate separated experiments are shown. * Significantly different from DW+ 10% MeOH based on the DMRT (Duncan’s multiple range test) (*p* < 0.05).

**Table 7:** Effect of isolated compounds from rice (*O. sativa*) on fresh weight of *E. oryzicola* after 7 days.

Sample Codes	Fresh Weight (mg)/Concentrations (µM)		
	100	250	500
1	199.77 ± 12.56 *	223.37 ± 16.21 *	220.03 ± 19.80 *
2	227.63 ± 31.79 *	222.47 ± 8.93 *	263.53 ± 42.43 *
3	211.17 ± 13.14 *	210.17 ± 12.83 *	210.87 ± 9.68 *
4	217.93 ± 18.71 *	197.90 ± 4.42 *	196.10 ± 31.41 *
5	275.47 ± 48.60	297.27 ± 13.46	287.63 ± 37.75
6	250.13 ± 3.94	264.23 ± 18.16	269.63 ± 12.69
7	251.93 ± 42.67	265.37 ± 39.94	263.10 ± 36.53
Pyributicarb	216.83 ± 15.47 *	254.87 ± 11.63 *	232.70 ± 30.74 *
DW+ 10% MeOH	311.57 ± 59.31		
DW	413.43 ± 45.87		

Mean values ± SD from triplicate separated experiments are shown. * Significantly different from DW+ 10% MeOH based on the DMRT (Duncan’s multiple range test) (*p* < 0.05).

**Table 8:** DPPH radical scavenging activity of the isolated chemicals.

Sample	IC_50_ (µM)	Correlation Coefficient (*r*^2^)
1	41.88 ± 1.21	0.99842
2	97.90 ± 0.98	0.99822
3	352.65 ± 2.56	0.95463
4	89.87 ± 2.32	0.99194
5	131.19 ± 1.56	0.99116
6	-	0.08371
7	-	0.43741

-: Not germination.
